# Efficacy and Safety of Mepolizumab (Anti-Interleukin-5) Treatment in Gleich’s Syndrome

**DOI:** 10.3389/fimmu.2018.01198

**Published:** 2018-05-29

**Authors:** Andrea Matucci, Francesco Liotta, Emanuele Vivarelli, Laura Dies, Francesco Annunziato, Marie Pierre Piccinni, Francesca Nencini, Sara Pratesi, Enrico Maggi, Alessandra Vultaggio

**Affiliations:** ^1^Department of Biomedicine, Immunoallergology Unit, AOU Careggi, Florence, Italy; ^2^Center for Research, Transfer and High Education DENOTHE, University of Florence, Florence, Italy

**Keywords:** Gleich’s syndrome, mepolizumab, anti-interleukin-5, biological agents, hypereosinophilia, angioedema

## Abstract

Gleich’s syndrome (GS) is characterized by recurrent episodes of angioedema, increase in body weight, fever, hypereosinophilia, and elevated serum IgM. The exact etiology remains unclear. Currently, the only treatment strategy is the administration of high dose of steroids during the acute phases. We report the case of a 37-year-old man suffering from GS with recurrent episodes of angioedema, fever, hypereosinophilia [6,000/mm^3^ (45%)], and high eosinophil cationic protein (ECP) (>200 μg/l), treated with oral steroids during the acute phase (prednisone 50–75 mg/day), the dose of maintenance being 25 mg/day. No monoclonal components were identified, and genetic tests exclude mutations including Bcr/Abl, JAK2 V617F, c-KIT D816V, and FIP1L1-PDGFRA. Using Luminex technology, we observed higher serum levels of interleukin (IL)-5, CCL2, and CCL11 during the acute exacerbations in comparison with the clinical remission phases though CCL11 did not achieve statistical significance. The flow-cytometric analysis identified a CD3+ CD8− lymphocyte population with high frequency of IL-4-, IL-5-, and IL-13-producing cells. No clinical benefit was observed after therapeutic strategies with imatinib, interferon-α, cyclosporine-A, and azathioprine. Due to high IL-5 serum levels, an intravenous treatment with anti-IL-5 monoclonal antibody mepolizumab (750 mg every 4 weeks) was started. A reduction in the rate of exacerbation phases/year (10 ± 3 vs 2 ± 1; *p* < 0.005), in the eosinophils count both in percentage (28.8 ± 12.8 vs 9.8 ± 3.9; *p* < 0.001) and absolute value (2,737 ± 1,946 vs 782 ± 333; *p* < 0.001) were observed as well as the ECP serum levels (132.7 ± 62.7 vs 21 ± 14.2 μg/l; *p* < 0.05). The daily dose of prednisone was significantly reduced (25 vs 7.5 mg). Any adverse effects were recorded. To the best of our knowledge, this case is the first report of the disease successfully treated with mepolizumab, and it could represent a novel therapeutic strategy in GS.

## Introduction

**Figure 1:**
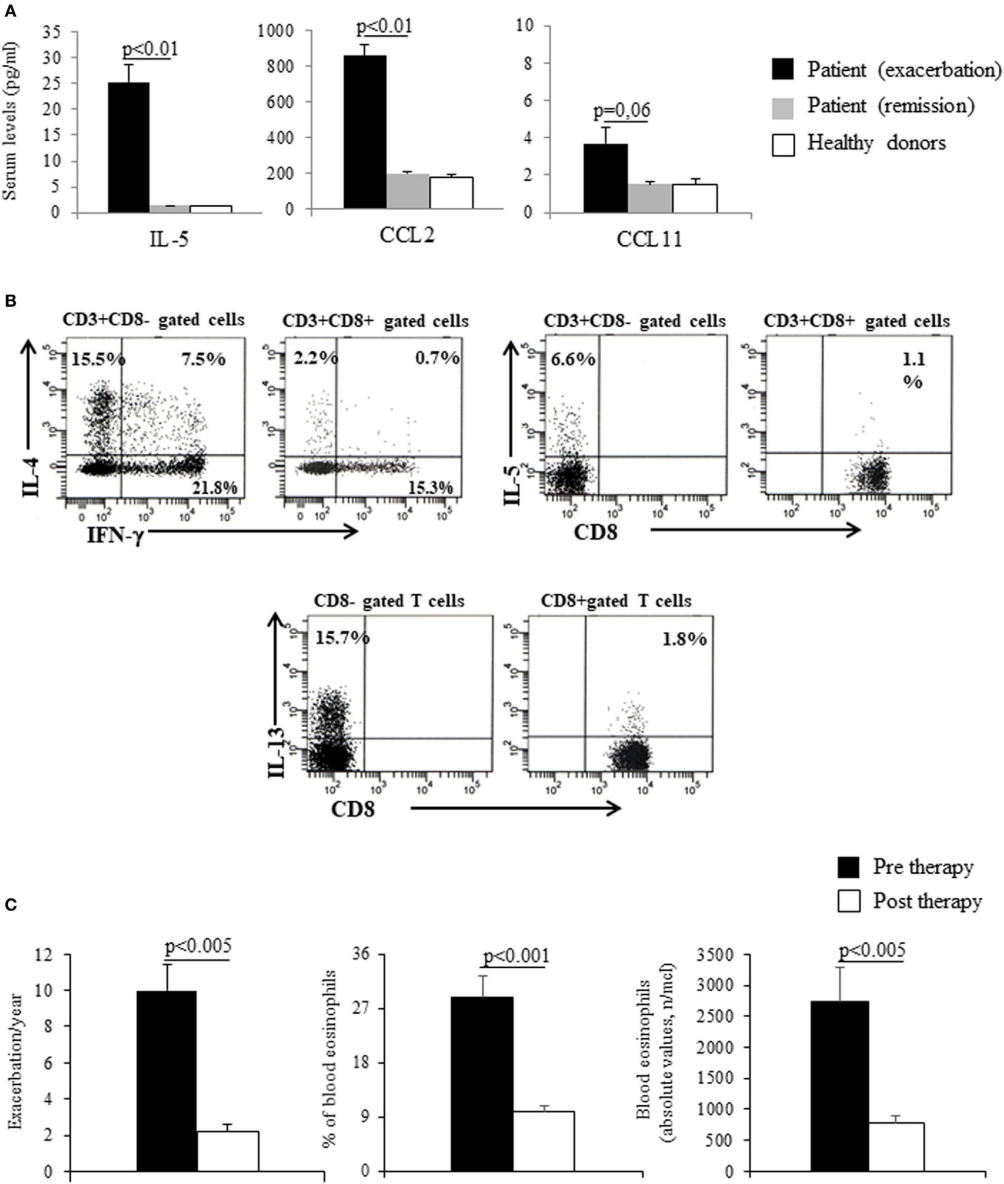
**(A)** Cytokines and chemokines serum levels during acute exacerbations and clinical remissions in the patient. Healthy donors’ values are reported as control. Mean values ± SEM are reported. **(B)** Cytofluorimetric analysis of cytokines production of peripheral blood T lymphocytes (CD3 gated) before treatment (peripheral blood mononuclear cells were polyclonal stimulated for 6 h in the presence of phorbol myristate acetate plus ionomycin, in the last 4 h brefeldin A was added to inhibit cytokines exocytosis. After stimulation, cells were fixed in formaldehyde, and cell membranes were permeabilized with saponin to permit the staining of intracellular cytokines with the appropriate fluorochrome-conjugated monoclonal antibodies. Samples were evaluated by BLSRDII flow-cytometer (BD-Biosciences, San Jose, CA, USA). At least 104 elements were acquired for each sample and analyzed by FacsDiva BD software. **(C)** Exacerbation/year and blood eosinophils (absolute value and percentage) before and after the treatment. Mean values ± SEM are reported.

We report the case of a 37-year-old man with a history starting at age 15 of recurrent episodes of angioedema of the head, trunk, and limbs, associated with myalgias, fever, hypereosinophilia [during an acute phase eosinophils had risen to 6,000/mm^3^ corresponding to 45% of circulating white cells and the eosinophil cationic protein (ECP) had risen to >200 μg/l], and also characterized by oliguria and weight gain (5–7 kg). The patient was being treated with high doses of oral steroids during the acute phase (prednisone 50–75 mg/day), the dose of maintenance being 25 mg/day. In October 2008, the patient was referred to our care and, according to classification criteria, was diagnosed with Gleich’s syndrome (GS). C1-esterase inhibitor levels and functional activity were in the normal range. At admission, the hemocromocytometric analysis showed hypereosinophilia (39% of WBC, 4,600/mmc) and increase of ECP (40 μg/l, n.v. <12 μg/l) and serum IgM [1,210 mg/dl (n.v. 40–230 mg/dl)], without any other alterations. No monoclonal components were identified. Total IgE was 134 kU/l (n.v. <85), and specific IgE for *Dermatophagoides pteronyssinus* (5.3 kUA/l; n.v. <0.10) was also observed. IgE for *Echinococcus* and *Aspergillus fumigatus* was not found. Parasitic infestations were excluded by serological tests and stool microscopic examination. A bone marrow biopsy showed “chronic pattern compatible with hypereosinophilic syndrome” while genetic tests to search for mutations including Bcr/Abl, JAK2 V617F, c-KIT D816V, and FIP1L1-PDGFRA were negative. Using Luminex technology, interleukin (IL)-5, CCL2, and CCL11 were repeatedly measured in serum during three different acute exacerbations and subsequent phases of clinical remissions. We observed higher levels of these cytokines during the acute exacerbations than in the clinical remission phases; at remission, values were similar to those observed in healthy controls (*n* = 5) (Figure 1A). As shown in Figure 1B, the flow-cytometric analysis of intracellular cytokine staining, upon polyclonal stimulation of peripheral lymphocytes collected during an exacerbation phase, showed a Th2-skewed cytokine profile. In particular, T helper (Th) cells, identified as CD3+ CD8− lymphocytes, showed high frequencies of IL-4-, IL-5-, and IL-13-producing cells, with normal values of IFN-γ. With regard to cytotoxic T cells, CD3+ CD8+ lymphocytes, they showed low proportion of IFN-γ-producing elements. In addition, the FACS analysis of TCR Vβ repertoire does not suggest a monoclonal expansion concerning the CD8+, CD4+ T cells, and CD3− CD4+ cells. For these latter cells, the analysis was performed at intracellular levels (data not shown). However, the existence of a very low percentage of monoclonal cell population among the CD3−CD4+ T cells cannot be completely excluded.

Previous therapeutic strategies, which had been performed in other centers, with imatinib and interferon-α had failed. We then proposed a therapeutic regimen with cyclosporine-A (3 mg/kg/day), as a steroid-sparing drug, but had to stop it after 6 months due its clinical inefficacy. We decided to start with azathioprine (100 mg/day), but the patient displayed a progressive increase of liver enzymes leading to therapy interruption after 2 months. The clinical manifestations were then managed with daily treatment with oral corticosteroids (25 mg/day) with increase of the dose (up to 75 mg/day) during the exacerbation phases.

Taking into account, the high serum levels of IL-5 shown in the patient and the role of this cytokine in eosinophil differentiation and survival, after obtaining written informed consent from the patient and the local Ethical Committee approval, in April 2011 we started intravenous treatment with anti-IL-5 monoclonal antibody mepolizumab (kindly supplied from GlaxoSmithKline) 750 mg every 4 weeks. After a follow-up period of 5 years, a reduction in the rate of exacerbation phases/year (10 ± 1.4 vs 2 ± 0.45; *p* < 0.005) and in the eosinophils count both in percentage (28.8 ± 3.6 vs 9.8 ± 1; *p* < 0.001) and absolute value (2,737 ± 5,536 vs 782 ± 104; *p* < 0.005) were observed (Figure 1C). Besides a reduction of the frequency of clinical attacks, the treatment with mepolizumab allowed a significant decrease of the severity of each exacerbation in terms of mean weight gain (5.3 ± 1 vs 1.3 ± 0.4, *p* < 0.0001) and in the daily maintenance dose of prednisone (25 vs 7.5 mg). Specifically, all the exacerbations experienced during the treatment have required short courses of medium doses of steroids (prednisone 15 mg/die for 5 days) to control symptoms. Furthermore, a statistically significant reduction of ECP serum levels (132.7 ± 62.7 vs 21 ± 14.2 μg/l; *p* < 0.05) measured in samples collected at exacerbations before mepolizumab treatment and during the patients’ visits at follow-up after the beginning of therapy.

Moreover, the frequency of IFN-γ-producing CD4+ T cells following polyclonal stimulation remained unchanged 10 months after the beginning of mepolizumab therapy, whereas the frequency of IL-5, IL-13 and in particular IL-4, producing CD4+ T cells was slightly reduced. With regard to CD8+ T lymphocytes, a clear increase of IFN-γ-producing cells was observed after therapy (Figure S1 in Supplementary Material).

The patient is still under treatment, and until now he has been submitted to a total number of 64 administrations, without any adverse effects. The patient has provided the written informed consent for the publication.

## Background

Gleich’s syndrome is a disorder characterized by recurrent episodes of angioedema, increase in body weight, fever, hypereosinophilia, and elevated serum IgM (1). Its exact etiology remains unclear, although it has been proposed that Th cells play a role by producing greater amounts of cytokines including granulocyte macrophage-colony-stimulating factor (GM-CSF), IL-3, IL-5, and IL-6 (2, 3). A specific treatment for GS is currently lacking, and the only strategy is the administration of a high dose of steroids during the acute phases of the disease. Recently, the availability of mepolizumab, a fully humanized IgG1 antibody specific for IL-5, has been approved for the treatment of severe eosinophilic asthma as well as eosinophilic granulomatosis polyangiitis (4, 5). Mepolizumab, administered intravenously at 750 mg every 4 weeks, has been demonstrated to be safe and effective as a corticosteroid-sparing agent in patients suffering from FIP1L1-PDGFRA negative hypereosinophilic syndrome (HES). No significant adverse events were observed in treated patients (6, 7).

## Discussion

To the best of our knowledge, this case is the first report of GS successfully treated with mepolizumab, even if the drug does not completely deplete eosinophils. IL-5 is a critical regulator of eosinophils and a therapeutic target in clinical conditions in which eosinophils are the main pathogenic cells, such as in HES. The demonstration of high serum levels of IL-5 in our patient during the exacerbation phases of the disease is in agreement with the therapeutic choice of using an anti-IL-5 antibody. Although a clonal expansion of CD4+ T cells has been shown in a proportion of patients suffering from GS (8), the production of high amounts of IL-5 can be sustained by a non-clonal T cell population, as in our case of GS. The therapeutic effect of mepolizumab is related to its ability to indirectly reduce eosinophil count; however, in our case, we observed the persistence of low levels of circulating eosinophils. These data likely reflect: (i) the incomplete neutralization of IL-5 which was overproduced and (ii) the presence of alternative pathways for eosinophil maturation and survival such as those induced by GM-CSF and IL-3 (9). On the other hand, mepolizumab is not expected to exert inhibitory effects on IL-5-producing T cells described in our case, and the small difference in the IL-5 expression after treatment does not allow us to draw any conclusions. Given that GS, like the other forms of HES, is a chronic disease, mepolizumab must be used over a long period; for this reason, and considering the prolonged eosinophil depletion, its safety profile must be confirmed. In experimental models, the eosinophil-deficient mice appear to display no overt changes in health, fecundity, ability to nurse, and vitality relative to normal mice (10). Concerning human experience, patients lacking eosinophils do not display any abnormalities related to eosinophil reduction (11). Furthermore, HES patients who have received mepolizumab for several years and who have not developed any specific set of adverse events confirm the safety of this monoclonal antibody (7).

In conclusion, mepolizumab has proved effective and safe in our patient and could represent a novel therapeutic strategy in GS even though additional studies are needed.

## Ethics Statement

This study was carried out in accordance with the recommendations of Internal Committee of AOU Careggi with written informed consent from all subjects. All subjects gave written informed consent in accordance with the Declaration of Helsinki. The protocol was approved by the Internal Committee of AOU Careggi.

## Author Contributions

AM, AV, and EM wrote, read, and provided bibliographic sources and approved the draft; FL and FA performed cytofluorimetric analysis; MP, FN, and SP contributed to cytokines’ detection and serological tests; LD and EV contributed to clinical support.

## Conflict of Interest Statement

AM and AV have served as investigators for GlaxoSmithKline. The remaining co-authors declare that the research was conducted in the absence of any commercial or financial relationships that could be construed as a potential conflict of interest.
