# Supplementary figures and images for: Efficacy and Safety of Mepolizumab (Anti-Interleukin-5) Treatment in Gleich’s Syndrome
Source: Front Immunol. 2018 May 29;9:1198. doi: 10.3389/fimmu.2018.01198 (PMC5986952; doi:10.3389/fimmu.2018.01198)

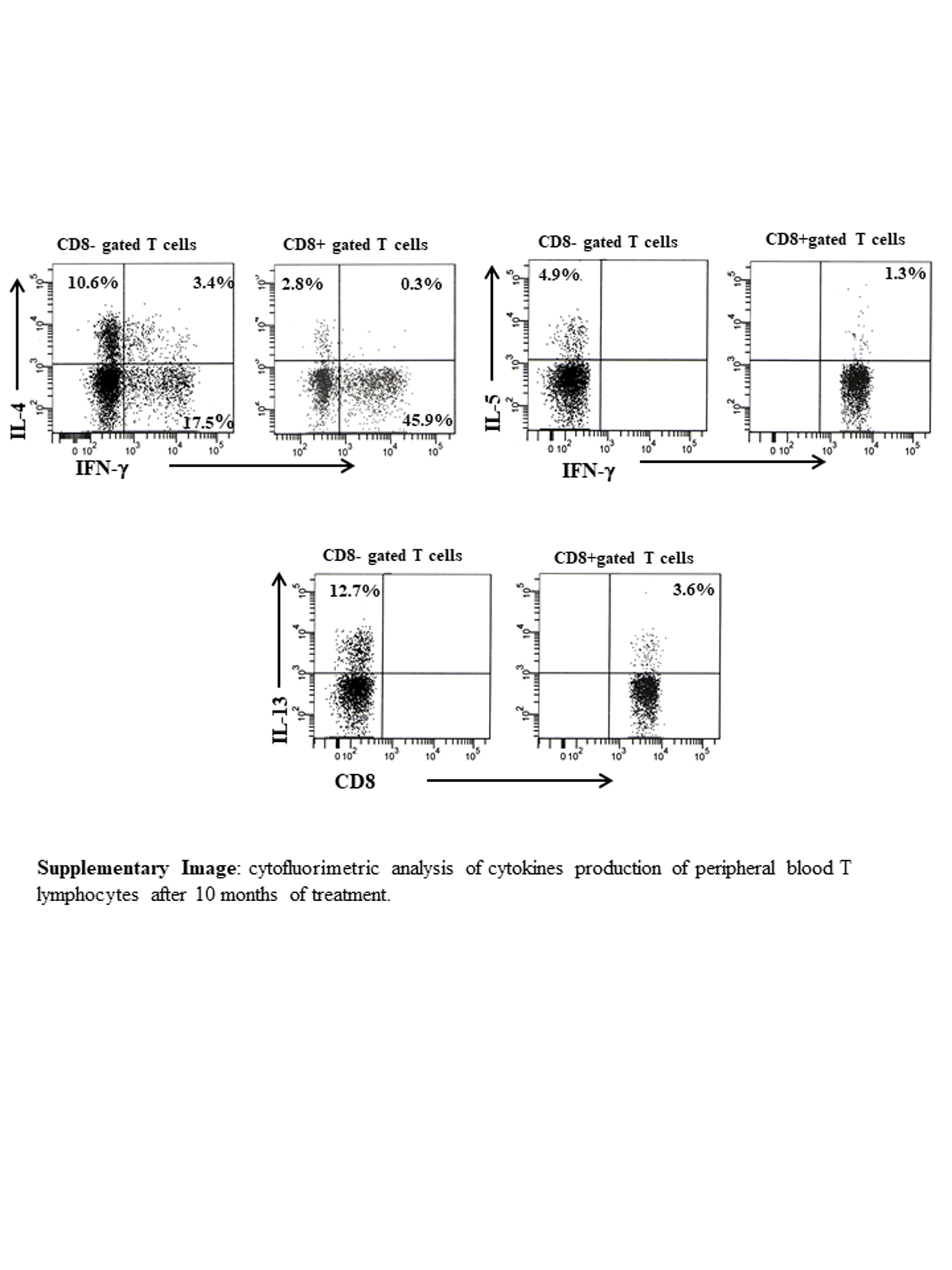

Supplement: Supplementary file 1 [file Image_1.tif]
